# Silver nanoparticles in sewage treatment plant effluents: chronic effects and accumulation of silver in the freshwater amphipod *Hyalella azteca*

**DOI:** 10.1186/s12302-018-0137-1

**Published:** 2018-02-13

**Authors:** Sebastian Kühr, Stefanie Schneider, Boris Meisterjahn, Karsten Schlich, Kerstin Hund-Rinke, Christian Schlechtriem

**Affiliations:** 1 Fraunhofer Institute for Molecular Biology and Applied Ecology IME, Auf dem Aberg 1, 57392 Schmallenberg, Germany; 2 Department Chemistry and Biology, “Ecotoxicology” Work Group, University of Siegen, 57068 Siegen, Germany

**Keywords:** Nanomaterials, Risk Assessment, Sewage treatment plant, Silver accumulation

## Abstract

**Background:**

Increasing amounts of engineered nanoparticles (NPs) in wastewater can reach the aquatic environment by passing through the sewage treatment plant (STP). NPs can induce ecotoxicological effects due to their specific chemical properties. However, their bioavailability and toxicity are potentially influenced by transformation processes caused by substances present in the STP, e.g., humic acids or sulfides. Due to the lack of a test system allowing to test NPs under realistic environmental conditions, we coupled two existing test systems, the activated sludge simulation test (OECD TG 303A 2001) and the chronic exposure test with the freshwater amphipod *Hyalella azteca* (Environment Canada 2013), to gain a test scenario that allows to consider the altered behavior and fate of NPs induced by the STP process. This should improve the environmental realism of the chronic exposure test with *Hyalella*. In the first study, we tested the STP effluent containing AgNPs. In the second and third study, tap water and control STP effluent were spiked with AgNPs and used as test media.

**Results:**

The chronic exposure studies with the freshwater amphipod *H. azteca* showed that the investigated AgNPs lose most of their toxicity while passing through the STP. Over all studies with total Ag concentrations ranging from 0.85 to 68.70 µg/L, significant effects of the AgNPs were only observed in the survival of test animals exposed to tap water containing the highest Ag concentration (62.59 µg/L). Accumulation of silver in the body of test animals was clearly dependent on the pretreatment of the AgNPs. Silver ions (Ag^+^) released from AgNPs are supposed to be the major pathway leading to body burden following exposure to test media containing AgNPs.

**Conclusion:**

The coupled test system is suitable for testing substances that can reach the environment via the STP effluent. The investigated AgNPs lose most of their toxicity while passing through the STP. Accumulation of silver in the animals exposed to the different treatments was apparent, whereby silver ions (Ag^+^) released from AgNPs were supposed to be the major pathway leading to body burden.

## Background

The production of engineered nanoparticles (NPs) occurs in large quantities and was estimated, by the European Commission, to be 11.5 million tons per year [1]. The wide application of silver nanoparticles (AgNPs) in textiles, packaging, personal care products, etc., especially due to their bactericidal effect, led to a steadily rising production volume of AgNPs. For the worldwide production of textiles, a usage of 1.2–36 tons/year is calculated [2]. For Europe, the application of AgNPs is estimated to be in a range of under 10 up to over 230 tons of silver per year [3–5].

By leaching of AgNP-coated products (e.g., textiles), AgNPs get into the wastewater [6]. Also, the industrial processing of NPs may lead to the release of AgNP into the sewage system [7]. Shafer et al. [8] have measured total silver (non-nanospecific) concentrations of up to 105 µg/L in the sewage influx of a sewage treatment plant (STP). It was shown that a large part of the AgNPs get retained in the sewage sludge during the wastewater treatment process [9]. However, a smaller part of the AgNPs can still reach the environment via the STP effluent [9–11].

Dissolved ionic silver (Ag^+^) is highly available for aquatic organisms [12]. However, different studies have shown that particulate silver (Ag) may be also bioavailable for aquatic organisms (e.g., [13–15]), also if presented as AgNPs. The uptake pathway differs depending on the organism and the form of the present Ag. For instance, Gomes et al. [16] showed in a study on Mediterranean mussel *Mytilus galloprovincialis* that the gills are the major organs for the uptake of dissolved metals, whereas NP aggregates are primarily taken up by the gut. NPs ingested by bivalves are also absorbed by the intestine through tubules of the digestive gland where they are taken up by endocytosis [17].

Ecotoxicological effects of AgNPs on aqueous organisms are well described. For fish, effects such as abnormalities of notochord and heart [15, 18–21], reduction of sodium uptake [22], respiratory stress [23] have been described with LC_50_-values starting at 34.4 µgAg/L [24–26]. Further studies describe lethal (e.g., [27]) as well as sublethal ecotoxicological effects on invertebrates such as decrease in motion activity, abnormal swimming behavior, and a reduced release of feces, which may have been caused by disruption of the digestive process, reduction of growth, and a decrease in the reproduction rate [24, 27, 28]. For algae, mostly sublethal effects resulting from an AgNP exposure, such as a decrease in chlorophyll, reduced photosynthetic yield, and an increase of lipid peroxidation, have been observed starting at concentrations of 1 mgAg/L [29–31].

The observed effects may be induced by interaction with the AgNPs or by Ag^+^ cations (Ag^+^) which are released from the AgNPs. The mechanisms of the ecotoxicological effects are well known and include oxidative and inflammatory processes, the release of genotoxic compounds [16], inducing mitochondrial dysfunction [18], destabilization of cell membranes, denaturation of proteins, and even the deterioration of DNA [16, 32]. DNA damages caused by Ag^+^ tend to be much higher than the damage caused by the AgNPs [16].

However, the toxic effects of AgNPs cannot be solely attributed to the release of Ag^+^ [9]. Asghari et al. [24] described the effect of AgNPs on the immobilization of *Daphnia magna* with an EC_50_ value around 47-fold lower, compared to the EC_50_ value of Ag^+^. Stronger effects of nanosilver in comparison to dissolved silver were also observed in a chronic toxicity test with *Daphnia magna* [27]. However, investigations on the alteration of gene expression in *Daphnia magna* following exposure to AgNPs showed a lower effect of AgNPs compared to silver ions presented in the form of silver nitrate [33].

It is assumed that the toxicity of AgNPs on aquatic organisms changes strongly upon passing through STPs, induced by the following mechanisms. Alterations of the particles can be caused by a number of possible transformation processes [9, 34–37]. Chemical transformations such as sulfidation, oxidation, or chlorination occur in environmental media and may reduce the solubility of AgNPs and the release of Ag^+^ and thus their toxicity [9, 34, 35, 38]. In contrast to this, Lee et al. [39] described the direct accumulation of sulfidized Ag from contaminated diets, in which sulfidized metals were supposed to be reduced in the acidic environment of the digestive system and thus Ag^+^ could become bioavailable.

In aquatic systems, AgNPs show a strong trend toward sedimentation. Therefore, in aquatic habitats the availability of AgNPs is supposed to be higher for benthic organisms [40]. In laboratory tests with aqueous media containing NPs, the freshwater amphipod *Hyalella azteca* is exceptionally well suited for bioavailability and toxicity testings due to the epibenthic lifestyle, short life cycle, and sensitivity for environmental chemicals [41]. *H. azteca* is a common test organism for aquatic toxicity tests in North America (ASTM) and has been often used in sediment studies on metals [42, 43], including bioaccumulation approaches [44]. The mechanisms of uptake and elimination of different metals in *H. azteca* are described [44–47]. The amphipod is the most sensitive benthic species regarding the exposure to Ag^+^ [5, 48] and allows the evaluation of the potential effects of AgNPs on lower trophic levels.

In this study, we investigated the effect of STP effluents containing AgNP on the growth and survival of *H. azteca*. Effluents from an activated sludge simulation test (OECD TG 303A) [49] with the silver nanomaterial NM 300K (JRC repository) were used to perform a chronic exposure test (Environment Canada 2013) [50]. Juvenile animals were exposed for 3 weeks to the effluents of a model STP spiked with AgNP. The same nanoparticle was also applied to control effluent from the model STP and to copper-reduced tap water. Both treatments were tested in further exposure studies with *H. azteca* to investigate the impact of STP treatment on the toxicological effect of AgNPs. At the end of the exposure studies, the Ag body burden, survival rate, and length of the test animals from the different treatments were determined. All animals were collected for analysis of total silver concentrations.

## Methods

### Handling of AgNP and preparation of the NP stock suspension

Studies were carried out with NM 300K, a test material representing AgNPs in the scope of the OECD Working Party on Manufactured Nanomaterials (WPMN) Sponsorship Program. The stock suspension of NM 300K contains 10.16% (w/w) AgNPs, has an average particle size of 15 nm, and is stabilized with agent NM-300 DIS, containing 4% (w/v) polyoxyethylene, glycerol, trioleate, and polyoxyethylene(20) sorbitan monolaurate (Tween 20) each [51]. For the preparation of the AgNP working suspension, applied to the model STP, NM-300K was diluted with ultrahigh-quality water, hand-shaken for 1 min, and sonicated for 15 min (640 W) (Bandelin, Sonorex) to disperse the AgNPs and to carefully homogenize the suspension.

### Model STP

A laboratory-scale STP simulation was carried out according to OECD TG 303A as described by Muth-Köhne et al. [51] to produce the test media for the toxicity tests [49]. Fresh active sludge was obtained from a municipal STP to inoculate the model STP units. STP simulation approaches were carried out with and without addition of AgNPs to generate Ag-STP and control STP effluents. The addition of AgNPs to the test system was achieved by supplementing the influent (test sewage) to reach a total Ag concentration of 560 µg/L. Effluents of the STPs were collected and used as test media in chronic toxicity tests with *H. azteca*. The control effluent was frozen and stored at − 20 °C to prevent alteration prior to the use as test medium supplemented with AgNPs. Samples of all media were collected and stored at − 20 °C for analysis of silver concentrations.

### Preparation of the test media

Test media prepared for the chronic toxicity tests with *H. azteca* are presented in Table 1. In the first study (Study I), model STP effluents were used as test media which were treated in two different ways to reduce toxic ammonium/ammonia concentrations in the water: (i) dilution of STP effluents with purified copper-reduced tap water (1:100) to decrease the concentration of ammonium and nitrite, (ii) aeration of the effluents for 24 h to oxidize the possible presently toxic ammonium and nitrite to less harmful nitrate. These treatments should represent the real environmental scenario.

**Table 1:** Media and preparation of the treatments in Studies I–III

Study	Treatment	Preparation	Content
Study I	C_I_	Copper-free tap water (mineral media)	–
	STP-C-d	Control STP effluent, diluted 1:100 with copper -free tap water	–
	STP-C-a	Control STP effluent, aerated for 24 h	–
	STP-Ag-d	AgNP-spiked STP effluent, diluted 1:100 with copper-free tap water	6.87
	STP-Ag-a	AgNP-spiked STP effluent, aerated for 24 h	68.70
Study II	C_II_	Copper-free tap water (mineral media)	–
	MM 1	Mineral media spiked with NM 300K	1.01
	MM 2	Mineral media spiked with NM 300K	4.49
	MM 3	Mineral media spiked with NM 300K	62.59
	MM Dis	Mineral media spiked with NM 300K dispersant (no AgNPs)	10
Study III	C_III_	Copper free tap water (mineral media)	–
	STP-C	Control STP effluent	–
	STP-C-Ag 1	Control STP effluent spiked with NM 300K	0.85
	STP-C-Ag 2	Control STP effluent spiked with NM 300K	3.03
	STP-C-Ag 3	Control STP effluent spiked with NM 300K	43.29
	STP-C-Dis	Control STP effluent spiked with NM 300K dispersant (no AgNPs)	10

Total Ag and dispersant concentration in µg/L

All media were stored up to 3 weeks at 4 °C prior to use. In the chronic exposure study (Study I), the Ag-STP effluent was compared with control STP effluent and a further control treatment which was prepared using copper-reduced tap water [52] only. In the other studies, copper-reduced tap water (Study II) and control STP effluent (Study III) were spiked with pristine AgNPs, allowing to elucidate the impact of the STP process (Study I) on the ecotoxicological effects of AgNPs. Three concentrations of NM 300K were tested (see Table 1). The concentrations of NM 300K supplemented to the test media reflected the measured concentrations of total Ag in the STP effluents (Study I). In addition to that one dispersant concentration of 10 µg/L was tested, equivalent to the dispersant content within the treatment with the highest AgNP concentration.

### *Hyalella azteca*

The freshwater water amphipod *H. azteca* used for the chronic exposure tests were raised in the laboratory of Fraunhofer IME, Schmallenberg. The strain was originally obtained from “Freds Haustierzoo” (Cologne, Germany). The stock culture was kept in 2 L flasks stocked with 30 adult amphipods each. The organisms were kept in reconstituted water containing bromide [45] and were fed 5 mg of ground fish feed (Tetramin^®^) three times a week to maintain optimal growth. A small piece of gauze (3 × 3 cm) provided a place of refuge. Three times a week each beaker was fed with 5 mg TetraMin^®^ (Tetra). Offspring were separated from the parent organisms once a week to be used in the exposure tests. Care was taken that only healthy amphipods free from observable diseases and abnormalities were used in these studies.

### Algae

During the exposure tests, animals were fed with the green algae *Desmodesmus subspicatus.* For the cultivation of the algae, culture media prepared with copper-reduced water were used [53]. Algae suspension (200 mL/filter) was filtered through glass microfiber filters (Whatman^®^ GF6, diameter 50 mm) to reach a dense layer of algae. The filters with the algae were frozen and stored at − 20 °C.

### Chronic exposure tests (21 days)

In Studies I–III test animals were exposed to different test media containing Ag, presumably as a mixture of dissolved (Ag^+^) and particulate Ag (AgNPs). The chronic exposure tests lasted 21 days. Every treatment (Table 1) consisted of 5 replicated test groups with 20 juvenile *Hyalella azteca* (7–14 days old) which were kept in 600 mL glass beakers filled with 500 mL test or control medium. The beakers were placed in a tempered water bath (25 ± 2 °C) and kept under a 16:8-h light/dark cycle with a light intensity of 500–1000 Lux during daytime. Each beaker was permanently aerated to ensure a sufficient oxygen supply (> 60%) during the studies. All beakers were covered with glass plates to avoid evaporation. Every 7 days, test media were replaced and media samples taken for analysis of total Ag concentrations. During media changes, all animals were taken out of the aged media and transferred into new beakers with fresh media. During the transfer, living animals were counted to determinate the survival rate in each test group. In all studies, a piece (1/8) of an algae-coated fiberglass filter was placed at the test start and following every change of media into each beaker to provide food to the test animals. During the studies, the test animals were grazing ad libitum the algal material from the filter surface. The concentrations of ammonia, nitrite, and nitrate were measured in the fresh and aged media by photometric measurements (NANOCOLOR^®^ 500D, Machery-Nagel). Three times a week, the pH of the test media and oxygen saturation were measured. At the end of the studies, the living animals were counted and killed by addition of ethanol (96%). All animals of a replicated group were photographed as a group. The pictures were used to determine the length of the animals by image analysis with the image processing program ImageJ^®^ (Wayne Rasband, National Institutes of Health). The animals’ length was measured along the carapace from the eyes to the uropod [41]. The animals collected during the studies were preserved in ethanol (70%) and stored at 4 °C until Ag content analysis.

### Determination of total silver concentrations in the aqueous test media

Total silver concentrations in the aqueous test media were determined by inductively coupled plasma optical emission spectrometry (ICP-OES) using an Agilent 720 ICP-OES (Agilent Technologies, Waldbronn, Germany) at wavelength of 328.068 nm. The instrument was calibrated before each measurement using a blank and calibration solutions covering a range between 0.1 and 75 µg/L. For each standard and sample, at least three measurements were taken and the mean was determined by the ICP-OES software. The calibration function was calculated using a linear regression algorithm of the ICP-OES software. A series of calibration standards were prepared by dilution of a stock solution of a commercially available certified Ag ICP standard containing 1000 mg/L Ag in 3% nitric acid (Merck, Darmstadt, Germany) into diluted aqua regia solution to adjust the standards to the matrix of the samples. Furthermore, for quality assurance purpose, to check the accuracy of the measurement, aqueous certified reference material TM 25.4 (Environment Canada; certified concentration 22.0 µgAg/L) was analyzed alongside the samples during each series. The analytical method was also verified using a multi-element Merck IV Standard, diluted to 50 µg/L. The silver recovery for the quality assurance samples was in the range of ± 15% of the certified value.

The test media samples were diluted with aqua regia at a ratio of 1:10 (1 mL sample diluted with 9 mL aqua regia) and digested using a microwave (Discover SP-D, CEM, Matthews, NC, USA; max temperature 175 °C, max pressure 25 bar). The digested solution was further filled up to a final volume of 15 mL with diluted aqua regia and analyzed by ICP-OES.

Nitric acid (69%, suprapure grade) was purchased from Roth (Karlsruhe, Germany) and hydrochloric acid (30%, suprapure grade) from Baker (Netherlands). Aqua regia was prepared by mixing HNO_3_ and HCl at a ratio of 1:3.

### Determination of total silver concentrations in samples of previously exposed *Hyalella azteca*

For the total Ag analysis of the test animals which were collected at the end of the chronic exposure studies, 15 mL of aqua regia (3:1; nitric acid: hydrochloric acid) was added to the samples prior to digestion in a microwave (Discover SP-D, CEM, Matthews, NC, USA; max temperature 175 °C, max pressure 25 bar). The heating time was set to 12 min with an initial energy of 200 W. The samples were heated up to 175 °C at a maximum pressure of 25 bar and the maximum temperature was kept constant for 10 min for the extraction in aqua regia and afterward cooled down for approximately 8 min to 20 °C. The digested samples were diluted up to 50 mL using nitric acid (0.5 M). The silver analysis was performed by inductively coupled plasma mass spectrometry ICP-MS (Agilent 7700 ICP-MS, Agilent Technologies, Waldbronn, Germany). Ag was quantified following the method described in [54] using the isotope ^107^Ag. A rhodium standard (Merck KGaA; CertiPUR) was applied as internal standard. All concentrations were evaluated using data of the no-gas mode of the analytical device.

### Data analysis

The percentages of the survival rates underwent an arcsine transformation using Excel^®^ (Mircosoft). All data were subjected to an analysis of variance (ANOVA) using the data analysis software Origin (OriginLab Corporation; OriginPro 2017G). Time weighted average concentrations (TWA) of Ag in test media of the different treatments were calculated for the experimental periods. Total Ag concentrations in the test animals were divided by the TWA concentrations of the media to gain an accumulation factor allowing to draw conclusions about the bioavailability of Ag in *H. azteca* exposed to the different treatments.

## Results

### Total silver concentrations in the test media

The total Ag concentrations measured in the test media are presented in Table 1. The total Ag concentration in the Ag-STP effluent (influent spiked with NM 300K to reach an influent concentration of 560 µg/L) was 68.70 µg/L (treatment STP-Ag-a). After diluting the medium with copper-reduced water (1:100), the measured total Ag concentration in the diluted Ag-STP medium (STP-Ag-d) was 6.87 µg/L.

In Studies II and III, the media were spiked with NM 300 K to reach in the highest treatments (MM 3 and SPT-C-Ag 3) test concentrations comparable to the undiluted Ag-STP treatment STP-Ag-a.

In Study II, where AgNPs were spiked to copper-reduced tap water, the TWA of total Ag in the test media ranged from 1.01 µg/L (MM 1) to 62.59 µg/L (MM 3).

Similar values for total Ag concentrations were shown in Study III, where AgNPs were spiked to control the STP effluent. Here, the Ag concentrations ranged from 0.85 µg/L (STP–C–Ag 1) to 43.29 µg/L (STP–C–Ag 3).

### Survival of *Hyalella azteca*

**Fig. 1:**
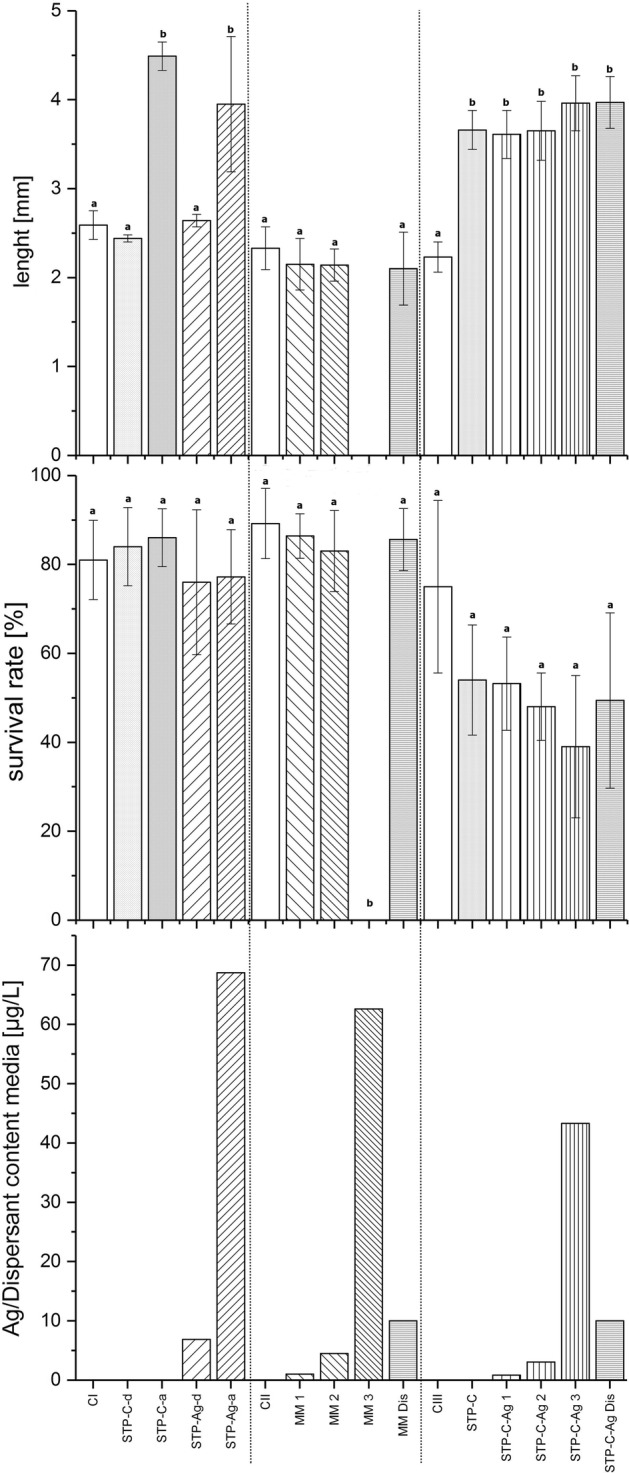
Results of chronic exposure studies (I–III). Measured Ag and nominal dispersant concentrations in µg/L, survival rate in %, and length (mm) of experimental animals

The survival rates of the animals are presented in Fig. 1. In Study I (effluent of the Ag-STP), the average survival rate in the treatments containing NM 300 K tended to be slightly lower (77% for STP-Ag-d and 76% for STP-Ag-a) compared to the water and STP effluent control treatment with survival rates > 80% at all concentrations. However, differences between the treatments were not significant.

In Study II (copper-reduced tap water spiked with AgNP), all animals died in the highest treatment (MM 3; Ag conc. 62.59 µg/L) until the end of the study. The observed differences between the control and the remaining Ag treatments with an average survival rate of 86% were not significant.

In Study III (AgNPs spiked into the effluent of the control STP), the highest survival rates were determined for the control treatments CIII and STP-C (75 and 54%) as well as for the STP control effluent spiked with the lowest Ag content (STP-C-Ag 1, 53%). No difference was observed between control effluent spiked with dispersant and all treatments containing different levels of NM 300K, but there were significant differences in the survival rates between those treatments and the control (C_III_). The lowest survival rate (39%) was observed in the highest treatment (STP-C-Ag 3).

### Length of *Hyalella azteca*

The length of all animals collected at the end of the studies was measured. The mean length determined for each treatment is presented in Fig. 1. In Study I (effluent of the Ag-STP), the longitudinal growth in the control treatment CI (Cu reduced water) was comparable (0.259 cm) to those treatments containing diluted STP effluent (STP-C-d, 0.244 cm; STP-Ag-d, 0.264 cm). The longitudinal growth in the non-diluted treatments (STP-C-a: 0.449 cm; STP-Ag-a: 0.395 cm) was almost two times and thus significantly higher than the diluted treatments (STP-C-d, STP-Ag-d) and CI. The length of the animals in the non-diluted treatments was comparable.

No significant difference in the longitudinal growth of the test animals was observed in Study II (copper-reduced tap water spiked with AgNP). In the control treatment (C_II_) with Cu-reduced water and the AgNP treatments, the mean length of the animals ranged from 0.21 (MM Dis) to 0.23 cm (C_II_). Due to the 100% mortality in treatment MM 3 no data for the longitudinal growth were measurable at the end of the study.

In Study III (AgNPs spiked into the effluent of the control STP), the mean length of the amphipods in the control treatment (C_III_) with Cu-reduced water was significantly smaller (0.223 cm) than those of the effluent control and the treatments (0.366 up to 0.397 cm). Within the different treatments, the longitudinal growth was similar. The supplementation of control effluent with dispersant had no effect on the growth of the animals.

### Total silver concentrations *Hyalella azteca*

**Fig. 2:**
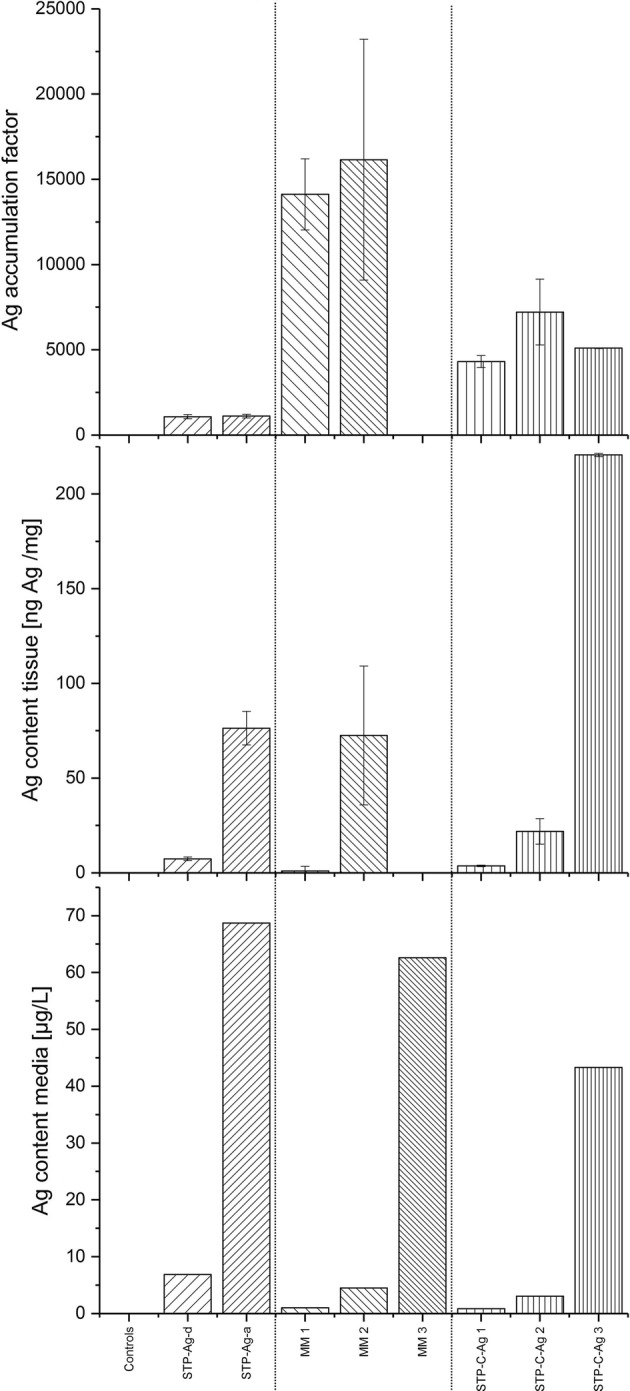
Ag content in media (µg/L), Ag content in animals (ng/mg) ± SD and accumulation of silver after 21 days of exposure. Ag accumulation factor = concentration in *Hyalella*/concentration in test medium

In Studies I–III, all replicates that were exposed to NM 300K showed detectable Ag concentrations (Fig. 2).

The measured body burden of total Ag in the animals seemed to correlate with the concentration of total Ag in the applied media. For example, the body burden of Ag in animals collected in Study I was 7.38 ng/mg (STP-Ag-d) and 76.38 ng/mg (STP-Ag-a), while the treatment concentrations of total Ag were 6.87 µg/L (STP-Ag-d) and 68.70 µg/L (STP-Ag-a). Due to the fact that no animals survived in the MM 3 treatment, only two values were measured in Study II. The Ag body burden of the animals in the remaining treatments MM 1 and MM 2 was 14.20 and 72.53 ng/mg, while the Ag concentration in the test media was 1.01 and 4.49 µg/L, respectively.

Similarly, in Study III, animals of STP-C-Ag 2 and 3 had an Ag body burden of 21.85 and 220.587 ng/mg after exposure to the test medium containing 3.03 µgAg/L and 43.29 µg/L, respectively. The lowest accumulation factors for total Ag estimated in the three studies was calculated for Study I (1075 and 1111 for STP-Ag-d and STP-Ag-a), while the highest factors were found in Study II (14,117 and 16,149 for MM1 and MM 2). Intermediate factors were estimated in Study III with 4307, 7214, and 5096 for STP-C-Ag-1, STP-C-Ag-2, and STP-C-Ag-3, respectively. One replicate of STP-Ag (Study I) and STP-C-Ag 3 (Study III) was excluded from the data analysis, following an outlier test (SQS 2013 Version 1.00 by J. Klein and G. Wachter).

## Discussion

### Chronic effects

Chronic effects of AgNPs in the effluent of a model STP, spiked to copper-reduced tap water, and spiked to STP control effluent on *H. azteca* were tested. The different exposure scenarios enabled us to estimate the influence of the wastewater treatment process on the toxicity and bioavailability of AgNPs present in the effluent. By discussing the chronic effects, it must be considered that the animals of each study originated from different batches. Therefore, the observed chronic effects were compared only within a single study.

The study has shown that the chronic effects of Ag on the freshwater amphipod *H. azteca*, which is known to be a sensitive benthic species for Ag exposure [48], are significantly reduced after passing the STP which could be explained by the transformations of AgNPs during the wastewater treatment process [9, 34, 35]. Exposure of test animals to STP effluents over 21 days resulted in an average survival rate of 80% in the control and all treatments. An NOEC for measured total Ag of 68.7 µg/L was determined in the first study. However, in a study on *Hyalella azteca*, Diamond et al. [55] estimated for Ag an NOEC of 0.9 µg/L and an LOEC of 1.9 µg/L for Ag after 21 days of exposure. This is similar to the results obtained in the second study (water spiked with NM 300K) where no effect on survival rates was observed in the two lower treatments with a total Ag concentration of 1.0 and 4.5 µg/L. The highest treatment with a measured Ag concentration of 62.6 µg/L, however, resulted in 100% mortality of the test animals at a medium concentration comparable to the NOEC determined with effluents obtained from the STPs. The differences in the toxicity of Ag observed in both studies might be explained by the transformation of the AgNPs while passing through the STP [9, 35]. Generally, metallic Ag is not persistent in aquatic systems under aerobic conditions with environmentally relevant pH values and will be oxidized to Ag^+^ [56]. The release of Ag^+^ in the test system following oxidation of the AgNPs is presumably the major mechanism leading to toxic effects [31, 57]. In contrast, organic compounds, present in the effluents, e.g., humic acids may reduce the toxicity by getting absorbed to the AgNPs surface as ligands [58]. Also, transformation processes such as sulfidation are known to lead to the passivation of the surface of AgNPs leading to a reduced release of Ag^+^ [56]. The passivation of the particle surface also prevents the generation of reactive oxygen radicals (ROS) at the particle surface, which are known to cause oxidative stress in exposed organisms [59, 60]. Dagon [61] and Bard et al. [62] described the transformation of Ag into Ag_2_S in the STP . This was confirmed also for AgNPs by Levard et al. [63] describing sulfidation as the major transformation process of AgNPs in STPs because of the high stability of Ag_2_S and the high amount of sulfide available in STPs and their sludge. Kaegi et al. [9] showed by transmission electron microscopy analysis and X-ray adsorption spectroscopy that Ag gets mostly adsorbed to the sludge as Ag_2_S. The toxicity reducing effect of sulfur compounds for Ag^+^-ions had also been described at environmentally relevant concentrations of sulfide, which reduced the acute toxic effects of Ag^+^ to *Daphnia magna* by about 5.5-fold [64, 65]. Considering these aspects, it could be hypothesized that AgNPs in the STP effluent (Study I) showed a higher degree of passivation most likely caused by sulfidation processes which led to a decreased toxicity compared to AgNPs spiked to Cu-reduced water (Study II).

The results obtained in the third study indicate that AgNPs spiked to STP control effluent were also transformed most likely, as described before, by sulfidation caused by the high amount of sulfide present in the effluent or detoxified by organic ligands like, e.g., humic acids [58]. This could explain why also in this study no effect on mortality could be observed. However, a small but non-significant trend to higher mortalities was still observed with increasing Ag concentrations. The lower survival rates, compared to the other studies, in all treatments and controls of Study III could be explained by the different condition of the animals used for this study, as mentioned above. It has been shown that the toxicity of NM 300K is mainly caused by Ag^+^ that is already present in the stock dispersion of this nanomaterial [66]. Zhao and Wang [27] described that there are no lethal effects in *Daphnia magna* when exposed to AgNPs when Ag^+^ is complexed with cysteine, while lethal effects were observed at lower Ag concentrations consisting of free Ag^+^. In this context, the small but non-significant trend to higher mortalities, observed in Study 3, might be explained by different levels of stock dispersion containing Ag^+^ which were added to the control effluent. With respect to study I, it can be assumed that Ag^+^ was mainly bound to the sewage sludge of the STP and was thus potentially leading to lower Ag^+^ concentrations in the effluent. This study investigated the dimension of toxicity induced by NM 300K in STP effluent that reaches the aqueous environment. In doing this, we did not differentiate between the concentrations of AgNPs and Ag^+^ in the media. The degree of transformation of AgNPs was not estimated. Further investigations are required to elucidate how alterations of AgNPs affect the toxicity of the NPs.

NM 300K contains a dispersant to improve the stability of the particles and which helps to prevent their settling or agglomeration. The potential influence of the dispersant on the performance of the test animals should not be overlooked, because dispersant concentrations in the test system increase along with increased AgNP concentrations. There are only a few studies available that discuss the toxicity of dispersants; however, it can be assumed that their impact is only limited. This was confirmed in Studies II and III, where the dispersant only was spiked to the mineral media (Study II) and control STP effluent (Study III). These treatments were supplemented with the highest concentration of the dispersant applied in Study I. The resulting survival rate of MM Dis in study II and ST-C-Ag-Dis of Study III was comparable with the control treatments of the respective studies.

### Effects on growth

Significant differences in the growth of the test animals over the test duration could be an indication for non-lethal toxic effects. However, the comparison of the length of the animals collected at the end of the study could exclude any growth inhibiting effects of AgNPs. In contrast, significant differences in the length of the animals were observed (Study I) between diluted (1:100 with copper-reduced tap water; STP-C-d, STP-Ag-d) and non-diluted STP effluent media (STP-C-a, STP-Ag-a).

Regarding the measured length of all animals in all three studies, we observe the same trend: significant higher lengths of the animals in treatments based on STP effluent compared to copper-reduced tap water only. In the treatments based on the diluted STP effluents (1:100 with copper-reduced tap water, STP-C-d, STP-Ag-d in Study I) and the tap water only treatments (all treatments of Study II, excluding C_II_), animals were around 40% smaller.

Those differences might be explained by the highest content of TOC measured in the treatments based on non-diluted STP effluents partly present as tiny organic particles which obviously provided a highly nutritious dietary supplement to the test animals. The STP effluents were not filtered before being used as test media to avoid modifications and reduction of the silver fraction. The Ag content of the organic material was not measured, but potentially contributed to Ag exposure as discussed below.

### Accumulation and body burden

The analysis of the total Ag concentrations in the animals and in the treatments showed that both seem to be positively correlated, with the highest test concentration always leading to the highest body burden. In all animals collected at the end of the three studies, a certain accumulation of silver was apparent. However, comparing the body burden of the test animals with the concentrations of their test media, accumulation factors were derived showing a differential uptake and accumulation of Ag depending on the history/pretreatment of the AgNPs. The results indicate that the transformation of AgNPs may also have an effect on the bioavailability of Ag. Accumulation factors of Ag calculated following exposure to supplemented mineral media were significantly higher compared to supplemented control STP effluent. The lowest accumulation factors were obtained for silver contained in the Ag-STP effluents. Cedervall et al. [58] mentioned that humic substances and proteins that are also found in STP effluents may change the bioavailability, toxic effects, and also the rate of bioaccumulation of NPs. It can be assumed that the AgNPs in the STP effluent had been mostly transformed to more stable compounds with low solubility and most of the released Ag^+^ would have been adsorbed to the sludge matrix [9, 34, 35, 63]. In comparison, AgNPs that were spiked to the diluted STP control effluent were supposed to be less sulfidized and therefore probably able to release more Ag^+^ which led to a higher accumulation of silver that we can observe by the higher accumulation in the treatments of Study III. Where AgNPs were spiked to Cu-reduced water (Study II), experimental conditions probably led to oxidation of AgNPs inducing a further release of Ag^+^, while the lack of organic ligands in the test medium maintained the constant availability of the dissolved ions.

AgNPs can be ingested by *H. azteca*, independent of their degree of transformation. It can be only speculated whether ingested AgNPs are bioavailable, e.g., by formation and adsorption of Ag^+^ in the gut, and how and to what extent they contribute to the body burden. Animals collected at the end of the study were not kept under control conditions for a certain time to evacuate their gut contents. The measured body burden of the animals may therefore partly represent ingested AgNPs. Further studies are required to elucidate the dietary uptake and accumulation of AgNPs by *H. azteca*, as well as to clarify the order of magnitude of gut contents contributing to the measured body burden.

### Suitability of the coupled test system

The studies allowed to assess the suitability of the coupled test system consisting of the activated sludge simulation test (OECD TG 303A 2001) combined with a chronic exposure test with the freshwater amphipod *H. azteca* (Environment Canada 2013) [49, 50]. The performance of chronic exposure studies with STP effluents provide a more realistic exposure scenario for NPs than the use of test media based on copper-reduced tap water supplemented with the test material. The chronic exposure test demonstrated that there were no toxic effects caused by the non-diluted STP effluent at the chosen concentrations.

During the test period, all essential water quality parameters such as oxygen saturation, the pH value or the concentrations of nitrate, nitrite and ammonium were in a range acceptable for *H. azteca*. However, if realistic exposure conditions are required STP effluents used in the laboratory should be diluted (e.g., 100-fold) to mirror the dilution of effluents discharged into receiving waters, like performed in this study.

The coupled test system showed to be suitable for testing NPs, but should also be suitable for testing organic compounds that also reach the environment by passing through the STP and which may also change their toxicity due to transformation processes. Animals were fed an algal diet to provide a sufficient supply of food during the study. However, the significant growth increment induced by highly nutritious organic particles suggest that a more balanced experimental diet should be used to feed the test animals [38, 52, 63, 64].

## Conclusions

The chronic exposure studies with the freshwater amphipod *H. azteca* show that AgNPs lose most of their toxicity while passing through the STP. In all animals collected at the end of the three studies, a certain accumulation of silver was apparent. Accumulation of silver ions (Ag^+^) released from AgNPs are supposed to be the major pathway leading to body burden following exposure to test media containing AgNPs. Further studies are required to further elucidate the bioavailability and bioaccumulation of silver following the ingestion of pristine, transformed, or agglomerated AgNPs.

## Abbreviations

Ag^+^: silver (I) ion
AgNPs: silver nanoparticles
ANOVA: analysis of variance
DNA: deoxyribonucleic acid
EC_50_: half maximal effective concentration
ICP-MS: inductively coupled plasma mass spectrometry
ICP-OES: inductively coupled plasma optical emission spectrometry
LOEC: lowest observed effect concentration
NOEC: no observed effect concentration
NP: nanoparticle
OECD: Organization for Economic Co-operation and Development
ROS: reactive oxygen radicals
STP: sewage treatment plant
TOC: total organic carbon
TWA: time weighted average concentration
